# Insights into the virome of *Hyalomma marginatum* in the Danube Delta: a major vector of Crimean-Congo hemorrhagic fever virus in Eastern Europe

**DOI:** 10.1186/s13071-024-06557-2

**Published:** 2024-11-22

**Authors:** Bianca Elena Bratuleanu, Delphine Chretien, Thomas Bigot, Beatrice Regnault, Philippe Pérot, Gheorghe Savuta, Marc Eloit, Sarah Temmam

**Affiliations:** 1 Regional Center of Advanced Research for Emerging Diseases, Zoonoses and Food Safety, “Ion Ionescu de La Brad” Iasi University of Life Sciences, Iași, Romania; 2 Pathogen Discovery Laboratory, Institut Pasteur, Université Paris Cité, 75015 Paris, France; 3 WOAH Collaborating Centre for Detection and Identification in Humans of Emerging Animal Pathogens, Institut Pasteur, Paris, France; 4 Bioinformatics and Biostatistics Hub, Institut Pasteur, Université Paris Cité, Paris, France; 5 UMR BIPAR, Laboratoire de Santé Animale, ANSES, INRAE, Ecole Nationale Vétérinaire d’Alfort, Maisons-Alfort, France; 6 Ecole Nationale Vétérinaire d’Alfort, University of Paris-Est, Maisons-Alfort, France

**Keywords:** Virome, Ticks, *Hyalommamarginatum*, Arboviruses, Romania, Surveillance

## Abstract

**Background:**

Ticks are significant vectors of pathogens, including viruses, bacteria, and protozoa. With approximately 900 tick species worldwide, many are expanding their geographical range due to changing socioeconomic and climate factors. The Danube Delta, one of Europe’s largest wetlands, is an ecosystem that, despite its ecological importance, remains understudied concerning the risk of introducing new tick-borne viruses. This region serves as a critical habitat for migratory birds, which can carry ticks over long distances, potentially introducing exotic tick species and their pathogens into the local ecosystem. *Hyalomma marginatum* ticks, the primary vector of Crimean-Congo hemorrhagic fever virus (CCHFV), are of particular concern due to their expanding presence in Europe and potential to spread other arboviruses. In addition to being the primary vector for CCHFV, *Hyalomma* sp. ticks are capable of transmitting other pathogens of medical and veterinary importance, including Dugbe virus, West Nile virus, African horse sickness virus, and Kyasanur forest disease virus. Therefore, it is essential to monitor the presence of *Hyalomma* sp. ticks while simultaneously surveilling arbovirus circulation in tick populations to mitigate the risk of arboviral outbreaks.

**Methods:**

In this work, we used an RNA sequencing technique to analyze the virome of *H. marginatum* ticks collected from the Danube Delta Biosphere Reserve, Romania, one of the major bird migration hubs from Africa to Europe.

**Results:**

Among the viral taxa detected in *H. marginatum* ticks, sequences belonging to Volzhskoe tick virus (VTV), Balambala tick virus (BMTV) and Bole tick virus 4 (BTV4) were identified. In addition, we report the first identification of a novel *Rhabdoviridae*-related virus, Hyalomma marginatum rhabdovirus (HMRV). No CCHFV or any CCHFV-related nairovirus were detected in this study.

**Conclusions:**

To summarize, detecting new viruses is essential for monitoring potential viral outbreaks. Our research expands the understanding of virus diversity in Eastern Europe, including the identification of novel viruses. This insight is crucial for monitoring viruses that may pose risks to both animal and human health, such as CCHFV.

**Graphical Abstract:**

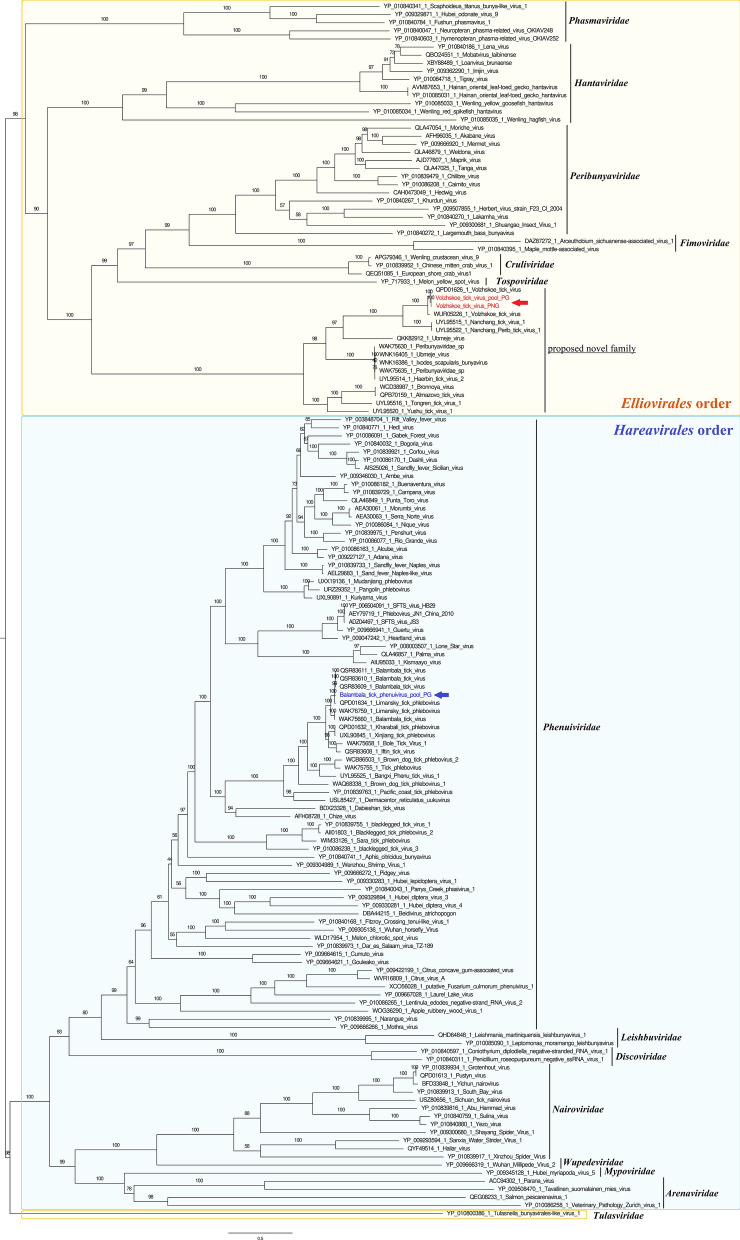

**Supplementary Information:**

The online version contains supplementary material available at 10.1186/s13071-024-06557-2.

## Background

Worldwide, there are around 900 tick species, with many capable of transmitting various pathogenic agents such as viruses, bacteria, and protozoa [1]. Changes in socioeconomic factors and climate patterns are driving shifts in the geographical distribution and seasonal patterns of several tick species, their vertebrate hosts, and the reservoirs of pathogens. Therefore, the implications of introducing and/or establishing new tick species along with their pathogens in regions previously unaffected by them is now an issue at the forefront [2, 3].

Despite its importance in terms of the risk of emerging tick-borne pathogens and despite recent studies describing the viral communities of ticks in the area [4–6], the Danube Delta ecosystem is still understudied, especially regarding the risk of introducing new viruses through migratory birds [7]. Ticks from the genus *Hyalomma* serve as an important example of this emerging risk [8]. In particular, *Hyalomma marginatum* is of considerable importance in veterinary and public health contexts due to its role as a main vector for Crimean-Congo hemorrhagic fever virus (CCHFV) [9]. While the virus has been detected in other tick genera (such as *Ixodes* sp. [10], *Haemaphysalis* sp. [10], and *Rhipicephalus* sp. [11]), *Hyalomma* sp. ticks remain the main vector of CCHFV worldwide [12]. Crimean-Congo hemorrhagic fever exhibits a high mortality rate in humans (around 30%) and poses considerable challenges in treatment, prevention, and control [13, 14]. In contrast, the infection is usually asymptomatic in domestic and wild animals, which may act as reservoirs of the virus. In Europe, the distribution of *H. marginatum* includes most of the Mediterranean and Balkan countries (France, Spain, Greece, Italy, Kosovo, Montenegro, Portugal, Croatia, Bosnia and Herzegovina, Albania, Bulgaria, Serbia, Ukraine, southern Russia, and Romania) [15], and migratory birds act as mechanical vectors for the spread of *Hyalomma* sp. ticks to new areas [16]. Endemic in Africa [9], Asia [17], and the Balkan countries [15], CCHFV is now expanding to Western Europe, where it was recently detected in Spain [12]. In France, the virus was recently discovered in *H. marginatum* ticks, without any human case yet [18]. In Romania, the virus was never detected in *Hyalomma* sp. or in any *Ixodidae* tick species, although it was reported in bordering countries including Hungary, Bulgaria, and Serbia [13, 14], and no acute infection was detected in ruminants although high seropositivity rates were previously reported [19].

Besides being the main vector of CCHFV, *Hyalomma* sp. ticks can also transmit other pathogens of medical and veterinary significance such as Dugbe virus, West Nile virus, African horse sickness virus, and Kyasanur forest disease virus [20–23]. Therefore, monitoring the introduction of *Hyalomma* sp. ticks along with surveillance of the circulation of arboviruses in tick populations is critical for mitigating the risk of arboviral outbreak. The rise of viral metagenomics and metatranscriptomics over the past decade has revolutionized virus discovery, uncovering a notable and unexpected diversity of viruses carried by ticks [24]. For example, in 2015, viral metagenomics identified a significant monophyletic cluster of newly discovered viruses, the family *Chuviridae* [25], which was associated with *Dermacentor*, *Haemaphysalis*, and *Rhipicephalus* species ticks from the French Antilles, Thailand, China, Brazil, and Trinidad and Tobago [26–28]. Also, five novel clades of RNA viruses were detected, exhibiting such divergent RNA-dependent RNA polymerase (RdRp) domains that they could potentially be classified as new virus families or orders, and were tentatively designated as Yuevirus, Qinvirus, Zhaovirus, Weivirus, and Yanvirus [29]. Since these viruses have so far been found only in ticks, their ability to infect vertebrates remains unknown. The identification of a growing number of viruses from various tick species in recent years suggests that the current understanding of tick-borne viruses is still limited, highlighting the need for comprehensive and agnostic studies.

The aim of our study was therefore to (i) provide the first snapshot of the composition of viral communities carried by *H. marginatum* ticks sampled in the Danube Delta Biosphere Reserve (DDBR); (ii) undertake an in-depth characterization of key viruses of the virome of *H. marginatum* ticks as a prerequisite for the identification of novel arboviruses of medical and veterinary significance; and (iii) try to explain the discrepancy between high seroprevalence in ruminants and the absence of detection of CCHFV in ticks from DDBR. This is essential for enhancing the control and prevention of significant epidemics caused by tick-borne viruses.

## Methods

### Tick sampling and identification

Twenty-five adult ticks belonging to *H. marginatum* species were sampled from the environment and from small ruminants (sheep and goats) within the DDBR (Romania) in May 2021. Ticks were morphologically identified using standard morphological keys [30], and the identification was confirmed by the extraction of sequencing reads targeting the cytochrome c oxidase subunit I (*COI*) gene and their submission to the Barcode of Life Data Systems (BOLD) database, as described previously [5, 31]. In brief, trimmed reads were first mapped to the *Ixodidae* BOLD database, and then mapped reads were de novo assembled and contigs were submitted to the BOLD Identification System (https://www.boldsystems.org/index.php/IDS_OpenIdEngine). The identification was further confirmed by the nucleotide Basic Local Alignment Search Tool (BLASTN).

### RNA extraction and metatranscriptomics sequencing

Ticks were divided into two pools, depending on the feeding status (engorged and questing), with each pool containing 12 and 13 ticks, respectively. Ticks were washed and cut lengthwise, and pools of half ticks were crushed using the Minilys homogenizer (Bertin) and Tungsten beads (Qiagen). Total RNA was extracted from the supernatant using TRIzol Reagent (Invitrogen) and the RNeasy mini kit (Qiagen) according to the manufacturers’ recommendations.

The two pools of ticks were used for next-generation sequencing (NGS) library preparation using the SMARTer Stranded Total RNA-Seq Kit v3–Pico input mammalian (Clontech, TaKaRa Bio, San Jose, CA, USA). Libraries were sequenced on an Illumina NextSeq 500 system in a single-read 1 × 150 base pair format.

### Bioinformatics analyses

Raw reads were processed using the Microseek bioinformatics pipeline [32] for virus identification. This included quality check, read trimming, normalization, assembly into contigs, and open reading frame (ORF) prediction for both contigs and singletons. Taxonomic assignment of predicted protein sequences was achieved through three levels of BLAST-based querying using the specialized Reference Viral DataBase (RVDB) [33] followed by invalidation of positive hits against two generalist protein and nucleotide databases (National Center for Biotechnology Information [NCBI]/nr [non-redundant] and NCBI/nt [nucleotide], respectively). Results were reported as the last common ancestor (LCA) to ensure a more accurate taxonomic assignation of viral hits. The abundance of each viral taxon was determined as the percentage of nucleotides assigned to a given taxon compared to the total number of nucleotides assigned as viral within a sample. A cut-off of a minimum of five reads assigned to a specific viral taxon was applied for further mappings and phylogenetic characterizations. Table S1 presents the complete list of viral taxa identified in engorged and questing tick pools, including those below five reads.

Phylogenetic analyses of reconstructed viral genomes were performed at the protein level on the conserved non-structural RdRp with representative viral sequences from various orders/families. First, consensus genomes were generated by mapping back trimmed reads to assemblies, and the majority consensus (cut-off 50%) was obtained using the CLC Genomics Workbench (Qiagen). Alignments were then conducted using the MAFFT algorithm under the L-INS-i parameter [34], and the best amino acid substitution model, determined via ATGC Smart Model Selection using the corrected Akaike information criterion [35], was determined as LG+G for all phylogenies. Phylogenetic relationships between discovered viruses and the diversity of known viruses within the genus, family, order or class were reconstructed using the maximum likelihood (ML) method provided by the IQ-TREE program [36]. Nodal support was evaluated using the approximate Bayes parameter.

## Results

### *Hyalomma marginatum* virome overview

In this study we performed RNA sequencing analysis of *H. marginatum* ticks collected in the environment and on small ruminants from two locations within the DDBR representing distinct biotopes (rural and forest areas). The taxonomic assignation revealed that 0.05% and 0.01% of total sequences were assigned to viruses in engorged and questing ticks, respectively. Most of the sequences were assigned to the *Ixodidae* and likely corresponded to the transcriptome of the tick.

The classified viral sequences were distributed into nine taxa, with viral abundance varying based on the pool considered. Seven taxa were in common between engorged and questing tick pools (i.e., Bole tick virus 4 [BTV4], Volzhskoe tick virus [VTV], *Totiviridae*, *Orthomyxoviridae*, *Virgaviridae*, *Phenuiviridae*, and *Rhabdoviridae*), while two were specific for questing ticks (*Mimiviridae*, *Retroviridae*) (Table 1). For example, the relative abundance of viruses assigned as BTV4 or VTV was higher in engorged ticks (56.4% and 23%, respectively) than in questing ticks (3.09% and 3.56%, respectively). In contrast, *Phenuiviridae*-related viral sequences, provisionally assigned as Balambala tick virus (BMTV), were distributed differently, with higher abundance observed in questing ticks than in engorged ticks (9.83% and 0.01%, respectively).

**Table 1:** Relative abundance of viral taxa detected in *H. marginatum* ticks from Danube Delta Biosphere Reserve

*H. marginatum* engorged tick pool		*H. marginatum* questing tick pool	
Viral taxa	Relative abundance	Viral taxa	Relative abundance
Bole tick virus 4	56.4%	Bole tick virus 4	3.09%
Volzhskoe tick virus	23%	Volzhskoe tick virus	3.56%
*Totiviridae*	1.07%	*Totiviridae*	4.76%
*Orthomyxoviridae*	0.117%	*Orthomyxoviridae*	1.47%
*Virgaviridae*	0.03%	*Virgaviridae*	49.9%
*Phenuiviridae*	0.01%	*Phenuiviridae*	9.83%
*Rhabdoviridae*	18.9%	*Rhabdoviridae*	21.9%
		*Mimiviridae*	5.50%
		*Retroviridae*	5.01%

### Characterization of key viral species

Among the viral taxa detected, some of these deserve special attention as belonging to viral families, orders, or classes that comprise known arboviruses. Comprehensive phylogenetic analyses were conducted to characterize these viruses as a first step towards the prediction of their ability to infect vertebrate hosts. We detected viruses assigned to the family *Flaviviridae*, the class Bunyaviricetes, and the family *Rhabdoviridae*. A few viral sequences were closely related to an unclassified quaranjavirus of the *Orthomyxoviridae* family (Guangdong tick quaranjavirus), but because of low genome coverage in both pools of ticks, they were excluded from the analysis.

### Bole tick virus 4

**Fig. 1:**
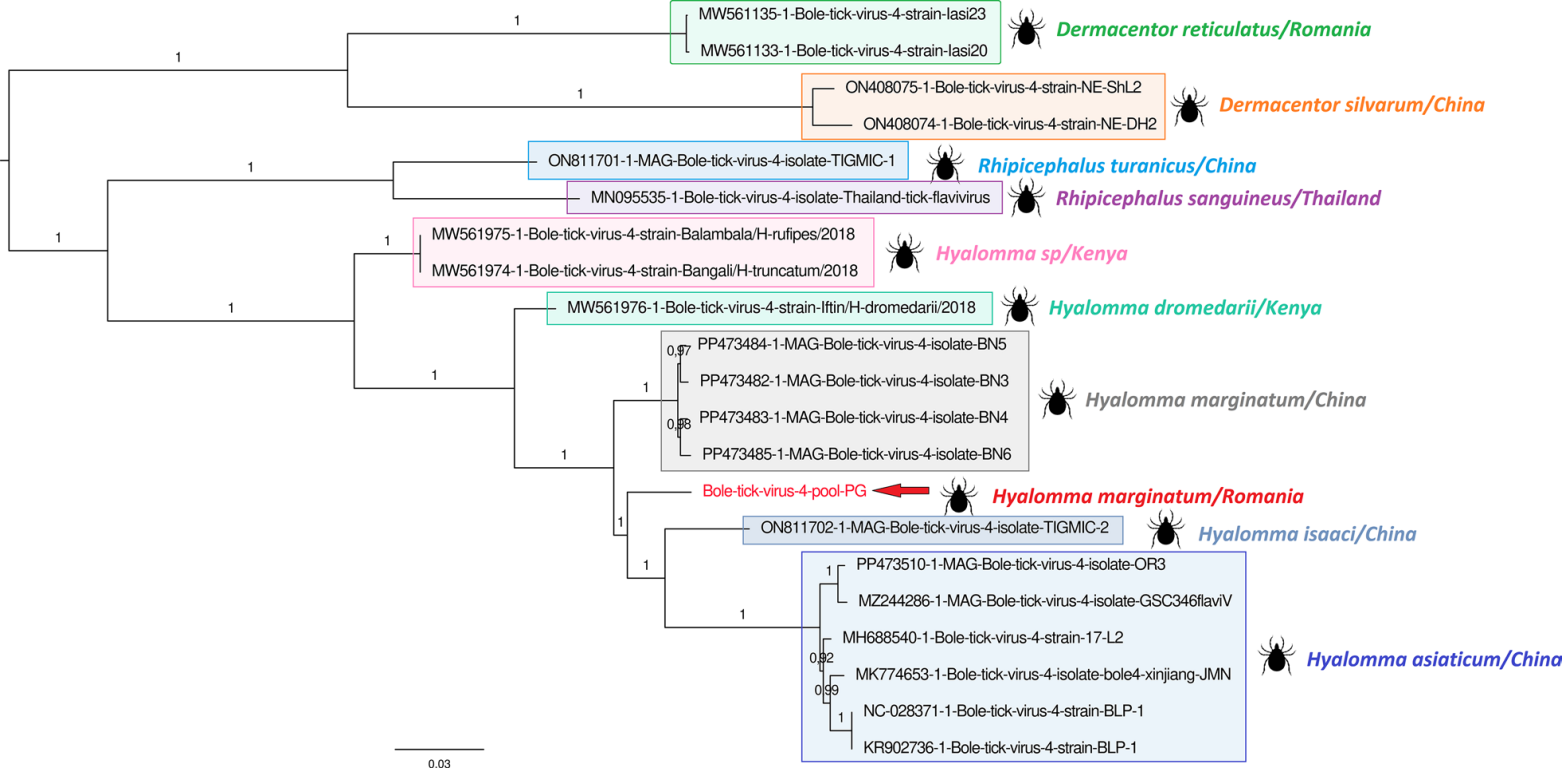
Phylogenetic analysis of the Romanian Bole tick virus 4 (BTV4) amino acid polyprotein in relation to BTV4 strains detected in different tick species. The BTV4 sequence is indicated in red

One of the most abundant viral sequences was assigned to BTV4, a virus not yet classified by the International Committee on Taxonomy of Viruses (ICTV) but phylogenetically linked to the family *Flaviviridae* [37]. Romanian BTV4 was identified in both engorged and questing ticks, presenting the highest abundance in the engorged pool. Genome coverage reached 100% and 45.8% for engorged and questing ticks, respectively. The virus identified in engorged ticks shared 97% amino acid identity with its closest tick-borne relative identified from China (WAK72328.1). Interestingly, *Hyalomma*-associated BTV4 was distant (below 90%) from other southern Romanian strains previously identified in *Dermacentor reticulatus*, *Rhipicephalus sanguineus*, and *H. punctata* ticks, suggesting the presence of several strains of BTV4 circulating in the same area. Phylogenetic analyses placed Romanian BTV4/engorged strain in a clade that includes other BTV4 tick-borne strains from southern Romania, China, and Thailand (Fig. 1). Of note, tick-associated BTV4 clustered differently depending on the tick genus and tick species considered, suggesting a strong association between the virus and its tick host.

### Volzhskoe tick virus

**Fig. 2:**
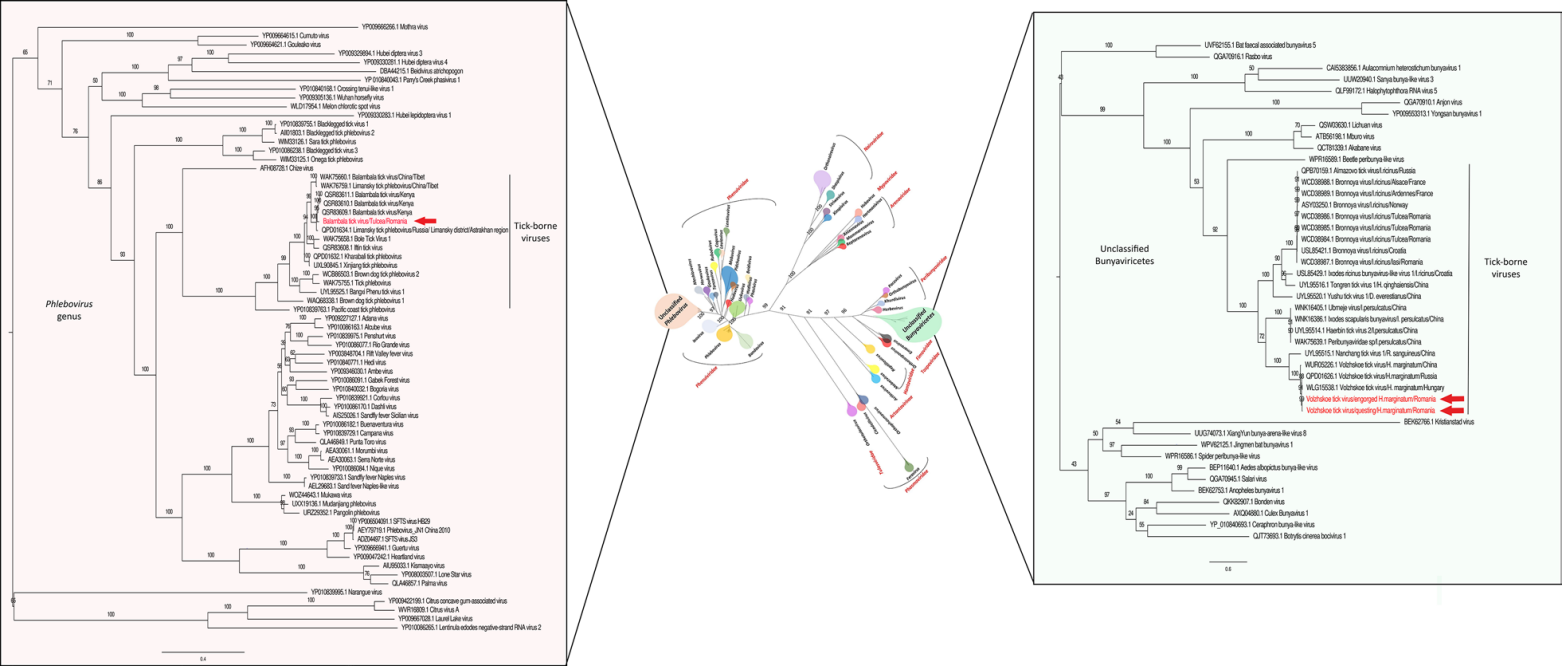
Phylogenetic relationship of Balambala tick virus (BMTV) and Volzhskoe tick virus (VTV) amino acid RNA-dependent RNA polymerase sequences identified in Romanian *H. marginatum* ticks with other viral families among the Bunyaviricetes. The BMTV and VTV sequences are indicated in red

The class Bunyaviricetes includes 15 classified families divided into the orders *Elliovirales* and *Hareavirales* [37]. We identified in *H. marginatum* engorged and questing tick viral sequences assigned to the order *Elliovirales* and closely related to VTV, with more than 85%amino acid identity with the prototype strain identified in *H. marginatum* ticks from Russia (QPD01626.1 and QPD01627.1). Romanian VTV strains were identified in both engorged and questing ticks and presented a horizontal genome coverage ranging from 65% to 100% (L segment) and from 57% to 100% (M segment), sharing high (99.6%) amino acid identity. The RdRp of the two strains of Romanian VTV showed an amino acid identity ranging from 97.9% to 98.2% with its closest tick-borne strain from Russia, while the glycoprotein presented a lower amino acid identity ranging from 87.5% to 96.95%. Phylogenetic reconstruction targeting the RdRp confirmed that Romanian VTV strains belong to the order *Elliovirales* and clustered in a clade that comprises other VTV strains recently detected in *H. marginatum* ticks from Hungary, Russia, and China. This clade is rooted by Nanchang tick virus 1, a tick-borne virus identified in *R. sanguineus* ticks from China. We propose that this clade, along with other tick-associated viruses identified in *Ixodes uriae* and *Ixodes persulcatus* from Sweden (Ubmeje virus), *I. persulcatus* from China (Haerbin tick virus 2), *Ixodes ricinus* from France, Romania, Croatia, Norway, and Russia (Bronnoya viruses), *Haemaphysalis qinghaiensis* from China (Tongren tick virus 1), and *Dermacentor everestianus* from China (Yushu tick virus 1), forming a distinct but well-supported clade, could constitute a novel family within the order *Elliovirales* (Fig. 2; Fig S1). This novel family is placed at the root of the *Peribunyaviridae* (comprising arboviruses), the *Fimoviridae* (infecting plants), the *Cruliviridae* (infecting crustaceans), and the *Tospoviridae* (infecting plants). Within this family, we observed that tick-borne viruses clustered differently depending on the tick species, Bronnoya-like viruses being associated with *I. ricinus* and distinct from *I. persulcatus* or *H. marginatum* viruses, suggesting a strong association and a possible restriction of this group of viruses with their tick host.

### Balambala tick virus

Within the class Bunyaviricetes, we detected in the order *Hareavirales* a high proportion of *Phenuiviridae*-related sequences belonging to the genus *Phlebovirus* in the pool of engorged ticks. Sequences were assigned to BMTV, a phlebovirus identified in numerous *Hyalomma* tick species worldwide. *Hyalomma marginatum*-associated Romanian BMTV presented a horizontal genome coverage ranging from 27.2% (S segment) to 97.7% (L segment). Pairwise comparison targeting the RdRp demonstrated that Romanian BMTV share between 97.6% and 98.7% amino acid identity with other BMTV strains, and a lower degree of conservation (between 91.8% and 87.5%) with a BMTV strain identified in Tibet in *Hyalomma isaaci* and with Limansky tick phlebovirus identified in *Hyalomma* ticks from Russia. Phylogenetic analysis placed Romanian BMTV within the group of unclassified phleboviruses, in a clade apparently restricted to tick-associated phleboviruses and sister clade of insect-associated phleboviruses such as Rift Valley fever virus and Toscana virus (Fig. 2, Fig S1).

### Hyalomma marginatum rhabdovirus

**Fig. 3:**
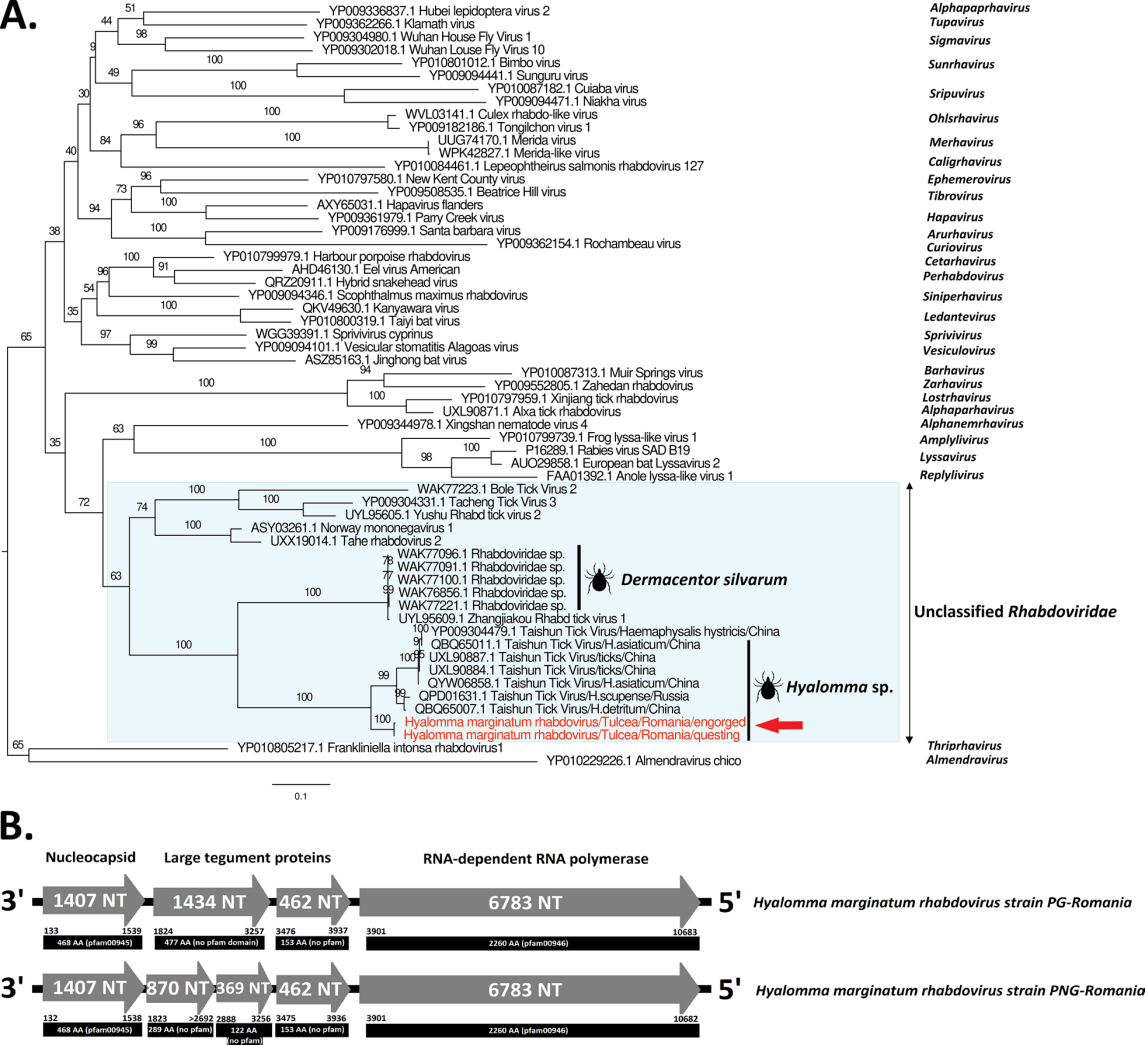
Phylogenetic relationship of Hyalomma marginatum rhabdovirus (HMRV) amino-acid polyprotein identified in Romanian ***H***. *marginatum* ticks with other viral genera among the *Rhabdoviridae* family (Fig. 3A) and the genomic structure of HMRV (Fig. 3B). The HMRV sequences are indicated in red

We identified two strains of a novel rhabdovirus, falling in the clade that could possibly constitute a novel genus within the family *Rhabdoviridae*, named Hyalomma marginatum rhabdovirus (HMRV). HMRV was detected in both engorged and questing ticks. In contrast to the classical rhabdovirus genome organization of N (nucleoprotein)–P (phosphoprotein)–M (matrix)–G (glycoprotein)–L (RdRp) genes, the complete genome of HMRV consists of four and five ORFs for HMRV/engorged and HMRV/questing viruses, respectively. These ORFs code for the nucleoprotein, two and three proteins of unknown function for HMRV/engorged and HMRV/questing viruses, respectively, and the RdRp (Fig. 3a). The pairwise comparison performed on the RdRp gene presented a high degree of amino acid conservation of HMRV strains between the two (99.6%). HMRV presented a horizontal genome coverage ranging from 98.8% to 100% and showed amino acid identity of 71.2% to 71.7% (RdRp) with its closest tick-borne relative identified in China (Taishun tick virus, GenBank accession no. QYW06858.1). Phylogenetic analysis placed Romanian HMRV strains in a clade encompassing tick-borne strains from China and Russia, recently detected in several species of *Hyalomma* and *H. hystricis* ticks (Fig. 3b), and sister clade of viruses identified in *Dermacentor silvarum*. In contrast to VTV, which exhibited a strong association with their tick species, Taishun tick-associated viruses exhibit a lower degree of association with *Hyalomma* species, as highly similar viruses were identified in *H. scupense* and *H. detritum* ticks (Fig. 3b).

## Discussion

In this research, we conducted RNA sequencing analysis aimed at detecting novel viruses associated with *H. marginatum* ticks, the main vector of CCHFV, collected from the DDBR in Romania, the second-largest wetland in Europe and the hub for many bird migrations. Despite its importance in terms of the risk of emerging tick-borne pathogens and despite recent studies describing the viral communities of ticks in the area [4, 5, 31], the Danube Delta ecosystem is still understudied regarding the potential introduction of new arboviruses through migratory birds. In addition, the discrepancy between the observed high seroprevalence of CCHFV in ruminants [19] and the absence of viral detection in ticks from DDBR is puzzling and needs to be explored, especially because the recent identification of CCHFV in various tick genera from Spain (*Hyalomma*, *Ixodes*, *Dermacentor*, and *Rhipicephalus*) [38, 39] and France [18] shows that the virus actively circulates among tick populations and expands its territory in Europe through bird migration.

Despite their importance in terms of human and animal health, the virome of *Hyalomma* sp. ticks is largely understudied. It has only been described in *H. dromedarii* ticks collected from camels in the United Arab Emirates, Saudi Arabia, and Kenya [40–42], and in *H. truncatum* and *H. rufipes* collected from camels in Kenya [42]. The first snapshot of the virome composition of *H. marginatum* ticks from Romania conducted here identified in high abundance viruses associated with the *Flaviviridae*, the *Elliovirales*, the *Phenuiviridae*, and the *Rhabdoviridae*. Similar viruses were identified in ticks from Kenya, United Arab Emirates, and Saudi Arabia, including BTV4 flavivirus and Balambala-like tick phleboviruses. Viruses were identified in both engorged and questing ticks and presented a high degree of genome conservation between the two pools of ticks (> 99% at the nucleotide level), but they were present in different abundance: for example, BTV4 flavivirus was dominant in engorged ticks while Balambala tick phlebovirus was dominant in questing ticks.

BTV4 is a novel virus provisionally classified into the *Flaviviridae* family but not yet recognized by the ICTV. It has been detected in multiple tick species around the world, as previously reported in *H. asiaticum* from China [43], *R. sanguineus* from Thailand, Romania, and Trinidad and Tobago [4, 44], and *D. reticulatus* and *H. punctata* from Romania [4], raising questions about its ability to infect vertebrate hosts. Indeed, phylogenetic analyses place BTV4 as a *Pestivirus*-like member of the *Flaviviridae*, and this genus is known to infect several mammals, including livestock (swine, sheep, goats, cattle) and wild animals (bats, rodents, giraffes, antelopes). Also, the detection of BTV4 in diverse tick species and the high degree of conservation of these strains across tick genera suggest that the virus could be acquired during tick co-feeding on the same vertebrate hosts, but further studies are necessary to confirm the origin and the pathogenicity of BTV4.

The order *Bunyavirales* was recently reclassified as the class Bunyaviricetes. Among this class, the order *Hareavirales* comprises the *Arenaviridae*, *Mypoviridae*, *Wupedeviridae*, *Discoviridae*, *Leishbuviridae*, *Nairoviridae*, and *Phenuiviridae* [37], with the *Phenuiviridae* comprising many tick-borne arboviruses. Tick-borne phleboviruses (TBPv) in particular are recognized as medically significant viruses due to the recent identification of new members in this family and their impact on human health. TBPv are primarily transmitted by *Ixodidae* ticks. Small mammals and birds have been proposed as potential reservoirs for these viruses [45], whereas humans and domestic animals are considered accidental hosts [46]. For example, one of the most important TBPv is severe fever with thrombocytopenia syndrome (SFTS) virus, causing epidemics in recent years [47]. In our work, we identified BMTV, a phlebovirus recently identified in numerous *Hyalomma* tick species worldwide (Ghana, Kenya, China, Russia) and in *Amblyomma* sp. ticks from Ghana (LC579820.1). *Hyalomma marginatum*-associated Romanian BMTV shared the same genomic structure as other novel phleboviruses, including BMTV strains identified in other *Hyalomma* species, that apparently missed the M segment coding for the viral glycoprotein [5, 25]. This absence of a segment could be either due to the existence of a yet unknown virus that complements the replication cycle of BMTV through co-infection, or simply because of a low level of genome conservation that prevents its identification with classical BLAST-based tools. Phylogenetic analyses placed BMTV in a clade apparently restricted to ticks, suggesting a possible specialization of this virus to its tick host. Its ability to infect vertebrate hosts along with its belonging to the core virome of *Hyalomma* sp. ticks is still unknown.

Within the class Bunyaviricetes, among the order *Elliovirales*, we identified VTV, which forms a distinct Romanian clade, separated from strains found in Hungary, Russia, and China. They are included in a distinct and well-supported group of viruses, which we propose could constitute a novel family within the order *Elliovirales* that comprises tick-associated viruses identified in *R. sanguineus*, *H. qinghaiensis*, *D. everestianus*, and *I. persulcatus* from China, in *I. uriae* and *I. persulcatus* from Sweden, and in *I. ricinus* from France, Romania, Croatia, Norway, and Russia (Bronnoya virus). This suggests a potential tick species and geographical specificity of these strains and the belonging of these viruses to the commensal flora of Romanian *Hyalomma* sp. ticks. To support this hypothesis, a previous study aimed at determining the ability of one of these viruses (Bronnoya virus) to infect ruminants was conducted in the same area of the DDBR as where the Romanian VTV was identified [6]. No sheep or goat serum was detected positive against Bronnoya virus, confirming the hypothesis that this novel viral family likely comprises tick-restricted viruses.

In addition to the discovery of Bunyaviricetes-related viruses, our study reports the identification of a novel *Rhabdoviridae*-related virus. Viruses of the family *Rhabdoviridae* can be transmitted through arthropod vectors such as mosquitoes, midges, sand flies, and ticks [48]. Over the last few decades, numerous novel rhabdoviruses have been reported worldwide [49–52], but few from tick vectors [53, 54]. In our study, HMRV were identified in both engorged and questing ticks. The virus clustered in a clade with other tick-associated viruses originating from different geographical biotopes, suggesting that there is no geographical specificity influencing the evolutionary history of these viruses, thereby strengthening the hypothesis regarding their vector specificity. Until now, these viruses have been found exclusively in ticks, so their capacity to infect multiple host species is questionable, despite the fact that *H. marginatum* is a two-host tick species that can infest a wide range of domestic and wild hosts (hares, hedgehogs, rodents, cattle, horses) or occasionally humans [55].

The last objective of our study was to identify, among the virome of *H. marginatum*, novel *Nairoviridae*-associated viruses that could explain through cross-reactivity the high seroprevalence observed against CCHFV in sheep and goats sampled in the DDBR and the absence of viral detection in ticks from the same area [19]. We based this hypothesis on the recent report of novel nairoviruses identified in non-*Hyalomma* ticks from the region, like Ixodes ricinus orthonairovirus and Sulina nairovirus in *I. ricinus* ticks [31] and Nayun tick nairovirus in *Rhipicephalus* sp. ticks [4, 31]. We did not identify any CCHFV, CCHFV-related, or *Nairoviridae* in our samples, suggesting that the maximal prevalence should be lower than 10% (*P* = 0.05). Although the sample size is too small to confidently capture a lower prevalence of the virus in ticks, previous studies conducted in Mauritania [56], Russia [57], Turkey [58], France [18], Pakistan [59], and Bulgaria [60] reported a prevalence of CCHFV of between 2% and 14.2% in *Hyalomma* sp. ticks. Our findings suggest that the virus is not yet circulating in the area, or is currently circulating but at a prevalence below the one observed in neighboring countries. Also, to confirm the absence of a novel tick-borne nairovirus able to infect ruminants and presenting with cross-reactive antibodies with CCHFV, enhanced sampling of ticks is needed in the region to cover the whole diversity of ticks present in the DDBR all year-round, and to exhaustively describe viral communities of ticks in the region. Such efforts are crucial to mitigating the risk of introduction and spread of tick-borne diseases in Europe through bird migration.

## Conclusions

In summary, the detection of new viruses is vital for monitoring potential viral outbreaks. Our study enhances the understanding of virus diversity in Eastern Europe, including the discovery of novel viruses. This knowledge is essential for monitoring viruses that may pose risks to both animal and human health, like CCHFV.

## Supplementary Information

Additional file 1.

Additional file 2.

## Data Availability

Sequence data reported in this manuscript were submitted to GenBank under the submission ID 2844888 (http://www.ncbi.nlm.nih.gov/Genbank) and the accession numbers are pending.
