# Supplementary material for: Insights into the virome of Hyalomma marginatum in the Danube Delta: a major vector of Crimean-Congo hemorrhagic fever virus in Eastern Europe
Source: Parasit Vectors. 2024 Nov 22;17:482. doi: 10.1186/s13071-024-06557-2 (PMC11585161; doi:10.1186/s13071-024-06557-2)
Supplement: Supplementary file 2 — Additional file 2. [file 13071_2024_6557_MOESM2_ESM.docx]

**Supplementary information**

**Fig S1.** Phylogenetic relationship of Balambala tick virus (BMTV) and Volzhskoe tick virus (VTV) RNA-dependent RNA polymerase identified in Romanian *H. marginatum* ticks with other viral families among the *Elliovirales* and *Hareavirales* orders

**Table S1.** Relative abundance of viral hits in *H. marginatum* questing and engorged ticks.

| Virus families and unclassified viruses | Relative abundance (%) | |
| --- | --- | --- |
|  | ***H. marginatum* questing ticks pool** | ***H. marginatum* engorged ticks pool** |
| *Virgaviridae* | 51.0 % | 0.0337 % |
| *Rhabdoviridae* | 22.0 % | 18.9 % |
| *Phenuiviridae* | 10.1 % | 0.0115 % |
| *Totiviridae* | 4.84 % | 1.06 % |
| Unclassified *Bunyavirales* | 3.65 % | 23.1 % |
| unclassified *Riboviria* | 3.14 % | 56.5 % |
| *Orthomyxoviridae* | 1.48 % | 0.170 % |
| *Retroviridae* | 1.27 % | 0.0875 % |
| *Iridoviridae* | 0.775 % | 0.117 % |
| unclassified *Negarnaviricota* | 0.216 % | 0.00594 % |
| *Coronaviridae* | 0.163 % | 0.0222 % |
| *Circoviridae* | 0.130 % | 0.00542 % |
| *Chrysoviridae* | 0.124 % | - |
| *Phycodnaviridae* | 0.108 % | - |
| Unclassified *Cressdnaviricota* | 0.106 % | - |
| *Parvoviridae* | 0.0887 % | 0.00400 % |
| *Picobirnaviridae* | 0.0814 % | - |
| unclassified viruses | 0.0683 % | 0.00335 % |
| *Secoviridae* | 0.0669 % | - |
| *Flaviviridae* | 0.0610 % | 0.00323 % |
| *Partitiviridae* | 0.0589 % | - |
| *Solemoviridae* | 0.0516 % | - |
| unclassified *Caudoviricetes* | 0.0516 % | 0.00542 % |
| *Caulimoviridae* | 0.0451 % | - |
| *Picornaviridae* | 0.0400 % | - |
| *Botourmiaviridae* | 0.0334 % | - |
| *Fiersviridae* | 0.0327 % | - |
| *Mimiviridae* | 0.0327 % | - |
| *Mitoviridae* | 0.0320 % | - |
| *Genomoviridae* | 0.0313 % | - |
| *Sedoreoviridae* | 0.0291 % | - |
| unclassified *Picornavirales* | 0.0283 % | - |
| *Luteoviridae* | 0.0203 % | - |
| *Dicistroviridae* | 0.0182 % | - |
| *Tombusviridae* | 0.0182 % | 0.00465 % |
| *Narnaviridae* | 0.0174 % | - |
| *Alloherpesviridae* | - | 0.00748 % |
| *Marnaviridae* | - | 0.00542 % |
